# Supplementary material for: ATR inhibitors as a synthetic lethal therapy for tumours deficient in ARID1A
Source: Nat Commun. 2016 Dec 13;7:13837. doi: 10.1038/ncomms13837 (PMC5159945; doi:10.1038/ncomms13837)
Supplement: Supplementary Information — Supplementary Figures [file ncomms13837-s1.pdf]

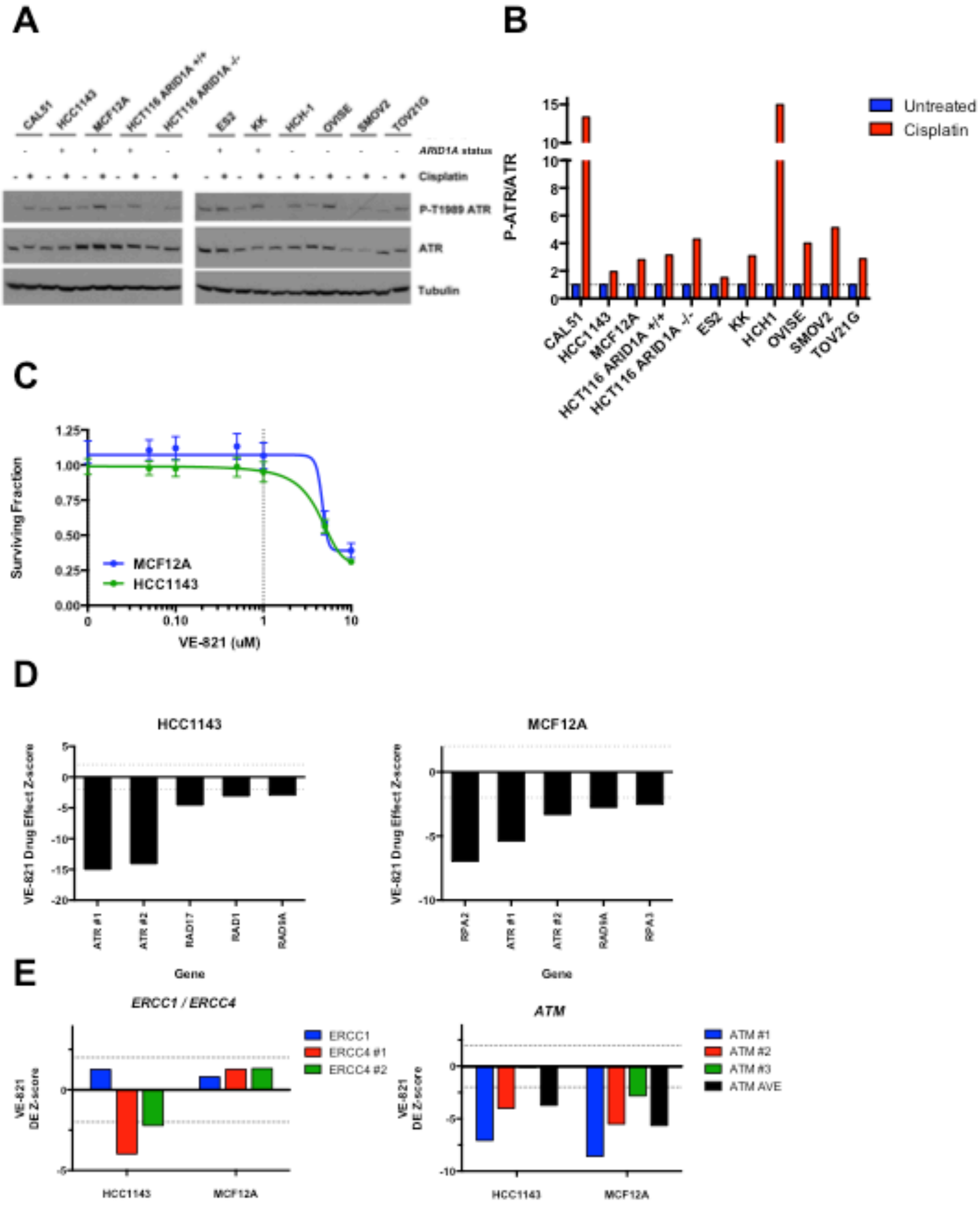
A
B
C
D
E
CAL51
HCC1143
MCF12A
HCT116 ARID1A +/+
HCT116 ARID1A -/-
ES2
KK
HCH-1
OVISE
SMOV2
TOV21G
ARID1A status
Cisplatin
P-T1989 ATR
ATR
Tubulin
P-ATR/ATR
Untreated
Cisplatin
HCH1
Surviving Fraction
MCF12A
HCC1143
VE-821 (uM)
VE-821 Drug Effect Z-score
ATR #1
ATR #2
RAD17
RAD1
RAD9A
RPA2
RPA3
Gene
ERCC1 / ERCC4
ERCC1
ERCC4 #1
ERCC4 #2
VE-821
DE Z-score
ATM
ATM #1
ATM #2
ATM #3
ATM AVE

Supplementary Figure 1. Additional RNAi screen data

A. Cisplatin induced ATR autophosphorylation. Western blot illustrating ATR and phospho-ATR (T1989) in cells exposed to 1 μM cisplatin for 24 hours prior to cell lysis. **B.** Cisplatin induced ATR autophosphorylation. Quantification of the level of cisplatin induced ATR autophosphorylation determined in (A). **C.** Sensitivity of MCF12A and HCC1143 to VE-821 in RNAi screen format assay. Dose response curves illustrating MCF12A and HCC1143 sensitivity to VE-821 following five days of drug exposure in a 384 well plate format. **D.** Bar chart illustrating VE-821 DE Z-scores from the high-throughput screen for genes implicated in ATR activation. ATR was represented two times in the RNAi screening library and both replicates are shown. **E.** Bar chart illustrating VE-821 DE Z-scores from the high-throughput screen for ERCC1, ERCC4 and ATM in HCC1143 and MCF12A. ERCC4 was represented two times in the RNAi screening library and both replicates are shown.

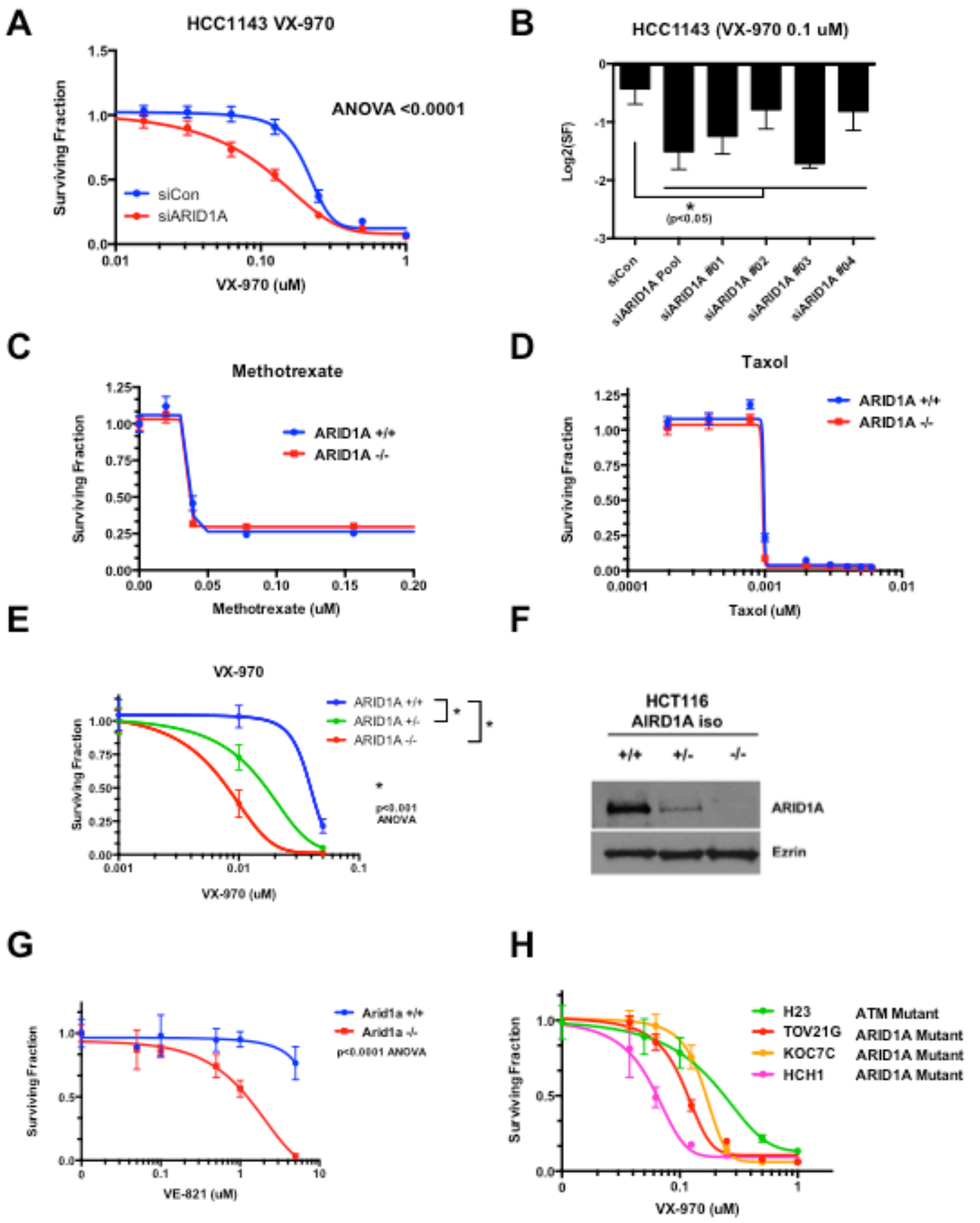

A
HCC1143 VX-970
ANOVA <0.0001
siCon
siARID1A
Surviving Fraction
VX-970 (uM)
B
HCC1143 (VX-970 0.1 uM)
Log2(SF)
*
(p<0.05)
siCon
siARID1A Pool
siARID1A #01
siARID1A #02
siARID1A #03
siARID1A #04
C
Methotrexate
ARID1A +/+
ARID1A -/-
Surviving Fraction
Methotrexate (uM)
D
Taxol
ARID1A +/+
ARID1A -/-
Surviving Fraction
Taxol (uM)
E
VX-970
ARID1A +/+
ARID1A +/-
ARID1A -/-
*
p<0.001
ANOVA
Surviving Fraction
VX-970 (uM)
F
HCT116
AIRD1A iso
+/+
+/-
-/-
ARID1A
Ezrin
G
Arid1a +/+
Arid1a -/-
p<0.0001 ANOVA
Surviving Fraction
VE-821 (uM)
H
H23
TOV21G
KOC7C
HCH1
ATM Mutant
ARID1A Mutant
ARID1A Mutant
ARID1A Mutant
Surviving Fraction
VX-970 (uM)

Supplementary Figure 2. Validation of ATR/ARID1A synthetic lethality

A. Dose response curves illustrating 384 well plate cell survival data from HCC1143 cells transfected with siRNA SMARTpool targeting *ARID1A* (red) or siCon (blue). Twenty-four hours after transfection, cells were exposed to VX-970 for five continuous days. Error bars represent SD from triplicate experiments. Survival curve siARID1A *vs.* siCon p-value <0.0001, ANOVA. **B.** Bar chart illustrating Log2 surviving fractions at 1 µM VX-970 in HCC1143 cells. Cells were transfected with individual *ARID1A* siRNA (#01, #02, #03 and #04). 24 hours after transfection cells were exposed to VX-970 (0.1 µM) or DMSO for five days. Viability was then estimated using CellTitre-Glo. Asterisk indicates statistically significant difference (Student's t-test, p<0.05). **C.** Dose response survival curves of HCT116 $ARID1A^{+/+}$ and $ARID1A^{-/-}$ cells exposed to methotrexate for five days prior to estimating cellular viability using CellTitre-Glo. **D.** Dose response survival curves of HCT116 $ARID1A^{+/+}$ and $ARID1A^{-/-}$ cells exposed to taxol for five days prior to estimating cellular viability using CellTitre-Glo. **E.** Dose response clonogenic survival curves of HCT116 $ARID1A^{+/+}$, $ARID1A^{+/-}$ and $ARID1A^{-/-}$ exposed to increasing concentrations of VX-970. Dose response curves from clonogenic assay are shown. Error bars represent SD (n=3), ANOVA p-value of <0.001 comparing $ARID1A^{+/+}$ vs. $ARID1A^{+/-}$ and $ARID1A^{+/+}$ vs $ARID1A^{-/-}$. **F.** Western blot of ARID1A protein expression in HCT116 *ARID1A* isogenic cells. **G.** $Arid1a^{-/-}$ mouse ES isogenic cells are sensitive to VE-821**.** Dose response curves from clonogenic assay are shown. Arid1a mouse ES isogenic cell lines were exposed to increasing concentrations of VE-821 for 10 days in a colony formation assay. Cells were then fixed, stained with SRB and colonies of 30 or more cells counted to estimate cellular viability compared to DMSO treated controls. Error bars represent SD from triplicate experiments. ANOVA p-value of <0.0001. **H.** ARID1A-deficiency induces comparable ATRi sensitivity to loss of ATM. Dose response curves for a panel of human tumor cell lines annotated by *ATM* or *ARID1A* mutation status. The indicated cell lines were exposed to VX-970 for five days prior to estimating cellular viability using CellTitre-Glo.

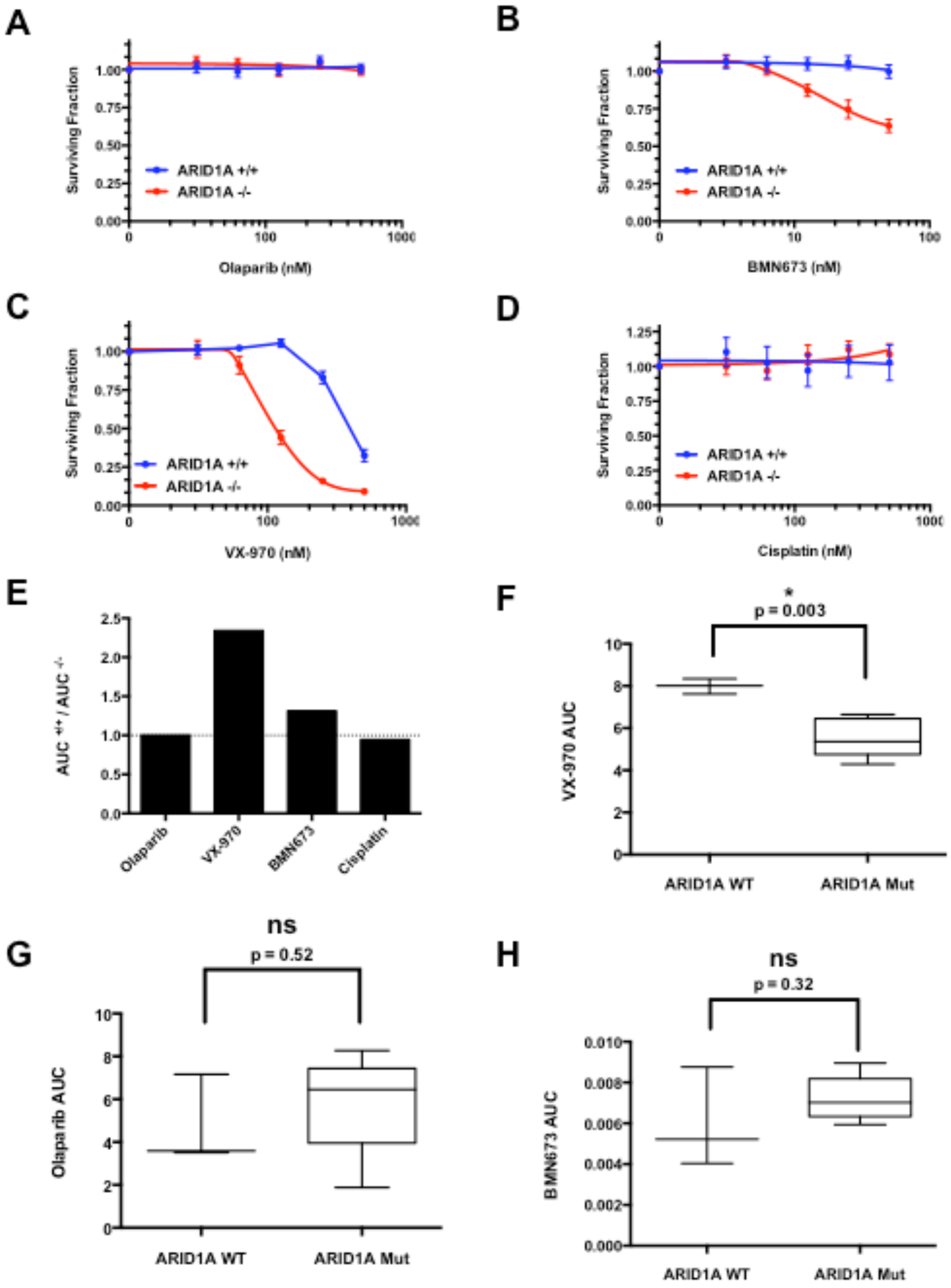
A
Surviving Fraction
ARID1A +/+
ARID1A -/-
Olaparib (nM)
B
BMN673 (nM)
C
VX-970 (nM)
D
Cisplatin (nM)
E
AUC +/+ / AUC -/-
Olaparib
VX-970
BMN673
Cisplatin
F
*
p = 0.003
VX-970 AUC
ARID1A WT
ARID1A Mut
G
ns
p = 0.52
Olaparib AUC
H
ns
p = 0.32
BMN673 AUC

Supplementary Figure 3. Olaparib, BMN673, VX-970 and cisplatin ARID1A$^{-/-}$ selectivity. HCT116 ARID1A isogenic (+/+ and -/-) cell lines were exposed to increasing concentrations of **(A)** olaparib, **(B)** BMN673, **(C)** VX-970 and **(D)** cisplatin. Following five days of drug exposure cellular viability was estimated by CellTitre-Glo. **E.** Bar chart represents the therapeutic window as represented by the fraction of $AUC^{ARID1A+/+}$ / $AUC^{ARID1A-/-}$ for the indicated drug. **F.** Box and whisker plots illustrating AUC data from ARID1A wildtype (WT, n= 3) or mutant (Mut, n=6) OCCC cell lines exposed to VE-821. Cells were exposed to VE-821 for five days prior to estimating cellular viability by CellTitre-Glo. Asterisk indicates statistically significant difference ($p<0.05$). **G.** As (F) for ARID1A wildtype or mutant (mut) OCCC cell lines exposed to BMN 673. **H.** As (F) for ARID1A wildtype or mutant (mut) OCCC cell lines exposed to olaparib.

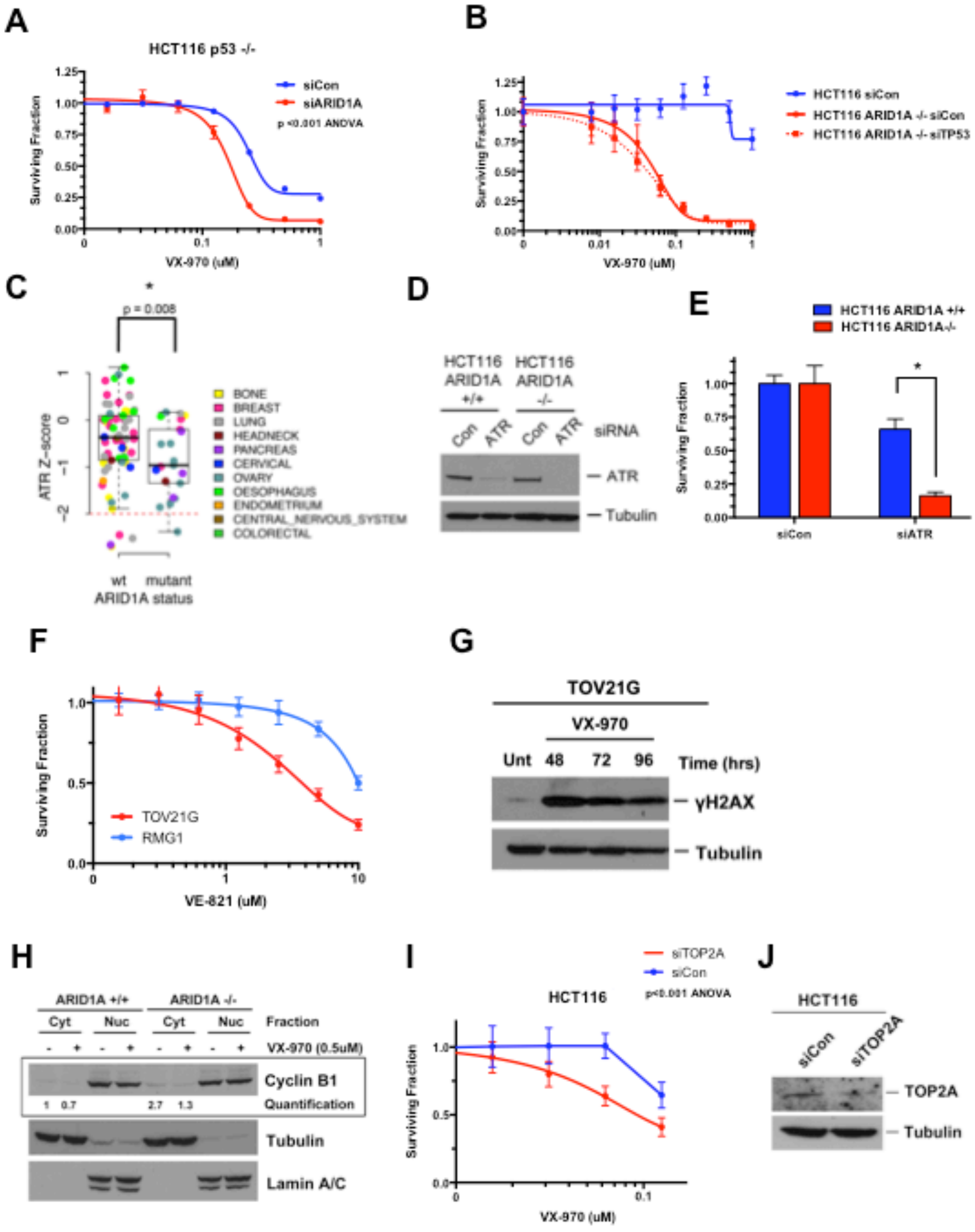

A
HCT116 p53 -/-
Surviving Fraction
VX-970 (uM)
siCon
siARID1A
p <0.001 ANOVA
B
HCT116 siCon
HCT116 ARID1A -/- siCon
HCT116 ARID1A -/- siTP53
C
p = 0.008
ATR Z-score
BONE
BREAST
LUNG
HEADNECK
PANCREAS
CERVICAL
OVARY
OESOPHAGUS
ENDOMETRIUM
CENTRAL_NERVOUS_SYSTEM
COLORECTAL
wt mutant
ARID1A status
D
HCT116 ARID1A +/+
HCT116 ARID1A -/-
Con ATR Con ATR siRNA
ATR
Tubulin
E
HCT116 ARID1A +/+
HCT116 ARID1A-/-
siCon
siATR
F
TOV21G
RMG1
VE-821 (uM)
G
TOV21G
VX-970
Unt 48 72 96 Time (hrs)
γH2AX
Tubulin
H
ARID1A +/+
ARID1A -/-
Cyt Nuc Cyt Nuc Fraction
VX-970 (0.5uM)
Cyclin B1
1 0.7 2.7 1.3 Quantification
Tubulin
Lamin A/C
I
siTOP2A
siCon
HCT116
p<0.001 ANOVA
VX-970 (uM)
J
HCT116
siCon siTOP2A
TOP2A
Tubulin

Supplementary Figure 4. Additional validation of ATR/ARID1A synthetic lethality

A. Dose response curves illustrating 384 well plate cell survival data from HCT116 p53-/- cells transfected with siRNA targeting *ARID1A* (red) or siCon (blue). Twenty-four hours after transfection, cells were exposed to VX-970 for five continuous days. Error bars represent SD. Survival curve siARID1A *vs.* siCon p-value <0.001, ANOVA. **B.** Dose response curves illustrating 384 well plate cell survival data from HCT116 and HCT116 *ARID1A*$^{-/-}$ cells transfected with siRNA targeting *TP53* (red dotted) or siCon (blue, red solid). Twenty-four hours after transfection, cells were exposed to VX-970 for five continuous days. Error bars represent SD. **C.** Z score box whisker comparison plot for 86 human tumor cell lines transfected with ATR siRNA. Cells lines were transfected with siRNA and cell viability was estimated five days later by CellTitre-Glo reagent. ATR siRNA Z-scores for ARID1A wildtype (n=65) and ARID1A mutant (n=21) were found to be statistically different (p=0.0084, median permutation test). **D.** Synthetic lethality with ATR siRNA HCT116 ARID1A +/+ and -/- cells were transfected with control siRNA or siRNA targeting ATR. Western blot demonstrating ATR protein level 96 hours following siRNA transfection. **E.** Bar chart illustrating cell viability 96 hours following siRNA transfection. Surviving fractions are normalized to control siRNA for each cell line. Asterisk indicates statistically significant difference (Student's t-test, p<0.05) between HCT116 ARID1A$^{-/-}$ and HCT116 ARID1A$^{+/+}$. **F.** Dose response curves for TOV21G (*ARID1A* mutant) and RMG1 (*ARID1A* wildtype) cells exposed to VE-821 for five days prior. **G.** Western blot illustrating γH2AX in TOV21G cells exposed to VX-970 (0.5 μM). Unt = untreated. **H.** Western blot showing Cyclin B1 levels in cytoplasmic (Cyt) and nuclear (Nuc) fractions of HCT116 ARID1A isogenic cells following exposure to VX-970 (0.5 μM) for 24 hours. Quantified levels of Cyclin B1 are shown. **I.** Silencing TOP2A sensitises HCT116 cells to VX-970**.** Surviving fraction curves from 384 well plate cell of HCT116 cells transfected with siRNA targeting *TOP2A* (red) or siCon (blue). Forty-eight hours after transfection, cells were exposed to VX-970 for five continuous days. Error bars represent SD. Survival curves siTOP2A *vs.* siCon p-value <0.001, ANOVA. **J.** Western blot illustrating TOP2A protein silencing.

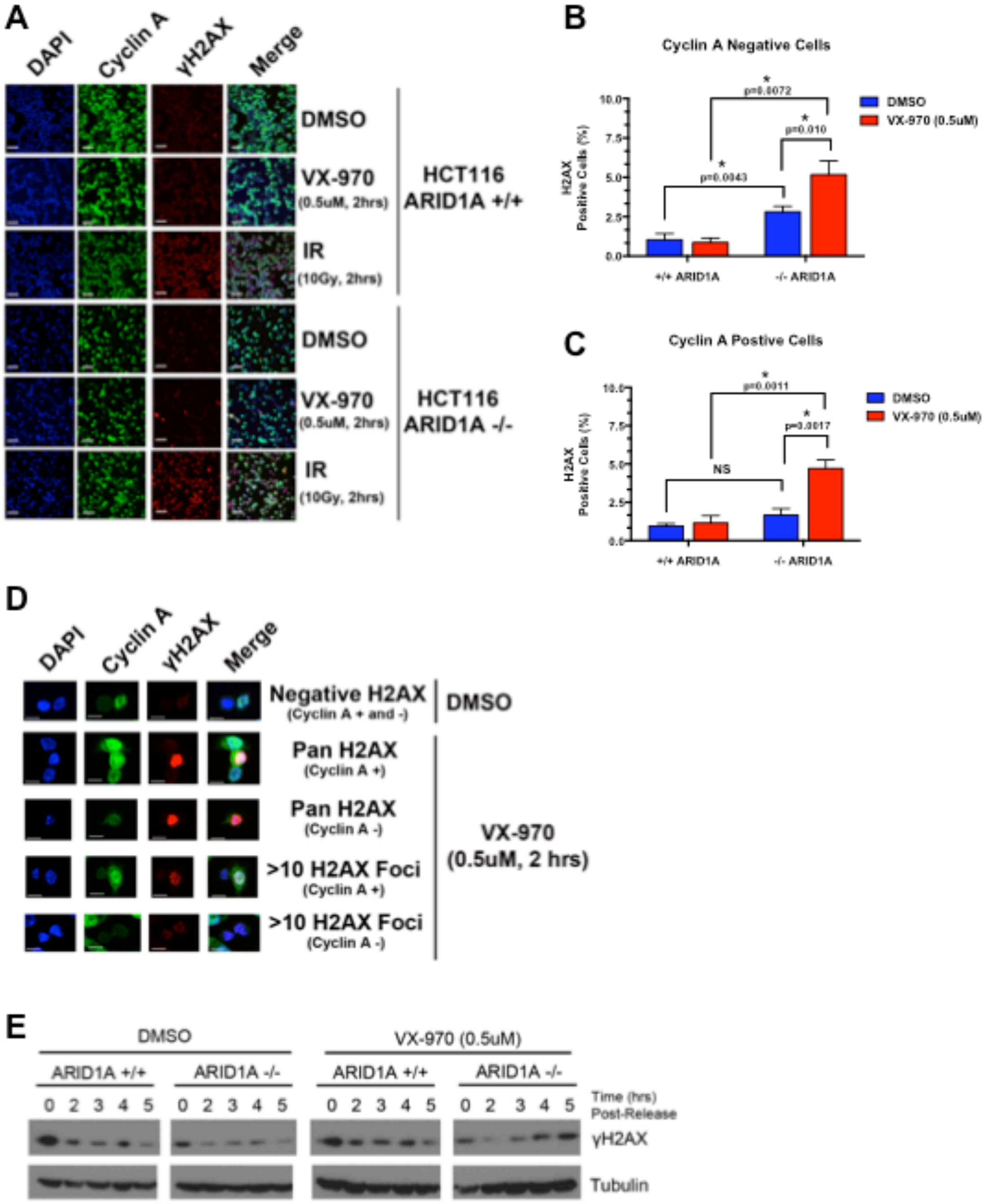
A
DAPI
Cyclin A
γH2AX
Merge
DMSO
VX-970
(0.5uM, 2hrs)
IR
(10Gy, 2hrs)
HCT116
ARID1A +/+
HCT116
ARID1A -/-
B
Cyclin A Negative Cells
H2AX
Positive Cells (%)
p=0.0072
p=0.010
p=0.0043
+/+ ARID1A
-/- ARID1A
DMSO
VX-970 (0.5uM)
C
Cyclin A Postive Cells
p=0.0011
p=0.0017
NS
D
Negative H2AX
(Cyclin A + and -)
Pan H2AX
(Cyclin A +)
Pan H2AX
(Cyclin A -)
>10 H2AX Foci
(Cyclin A +)
>10 H2AX Foci
(Cyclin A -)
VX-970
(0.5uM, 2 hrs)
E
DMSO
VX-970 (0.5uM)
ARID1A +/+
ARID1A -/-
Time (hrs)
Post-Release
0 2 3 4 5
γH2AX
Tubulin

Supplementary Figure 5. ATRi induced γH2AX in ARID1A$^{-/-}$ cells prior to mitotic entry

A. Representative immunofluorescence images of HCT116 *ARID1A*$^{+/+}$ and *ARID1A*$^{-/-}$ cells stained with DAPI, Cyclin A, γH2AX and a merged image, following treatment with DMSO, IR (10 Gy, 2 hours) and VX-970 (0.5 μM, 2 hours). Scale bar equals 75μM. **B.** Fraction of Cyclin A negative staining positive for γH2AX (>10 foci and/or pan nuclear staining) following DMSO or VX-970 (0.5 μM, 2 hours) treatment in HCT116 *ARID1A*$^{+/+}$ and *ARID1A*$^{-/-}$. Error bars represent SD for 3 biological replicates. No less than 200 cells were counted on four large field images for each replicate. P-values calculated using Student's t-test and * indicates statistical significance ($p<0.05$). **C.** Fraction of Cyclin A positve staining positive for γH2AX (>10 foci and/or pan nuclear staining) following DMSO or VX-970 (0.5 μM, 2 hours) treatment in HCT116 *ARID1A*$^{+/+}$ and *ARID1A*$^{-/-}$. Error bars represent SD for 3 biological replicates. No less than 200 cells were counted on four large field images for each replicate. P-values calculated using Student's t-test and * indicates statistical significance ($p<0.05$). NS indicates the comparison is not statistically significant ($p>0.05$ by Student's t-test). **D.** Representative images of the various classifications used to score cells as H2AX positive in panels B and C. Scale bar equals 6 μM. **E.** Western blot of cell lysates taken from the indicated time points from HCT116 *ARID1A*$^{+/+}$ and *ARID1A*$^{-/-}$ cells following release from synchronisation in G_1/early S phase by double thymidine block. Cells were released from thymidine block into media containing DMSO or VX-970 (0.5 μM).

A

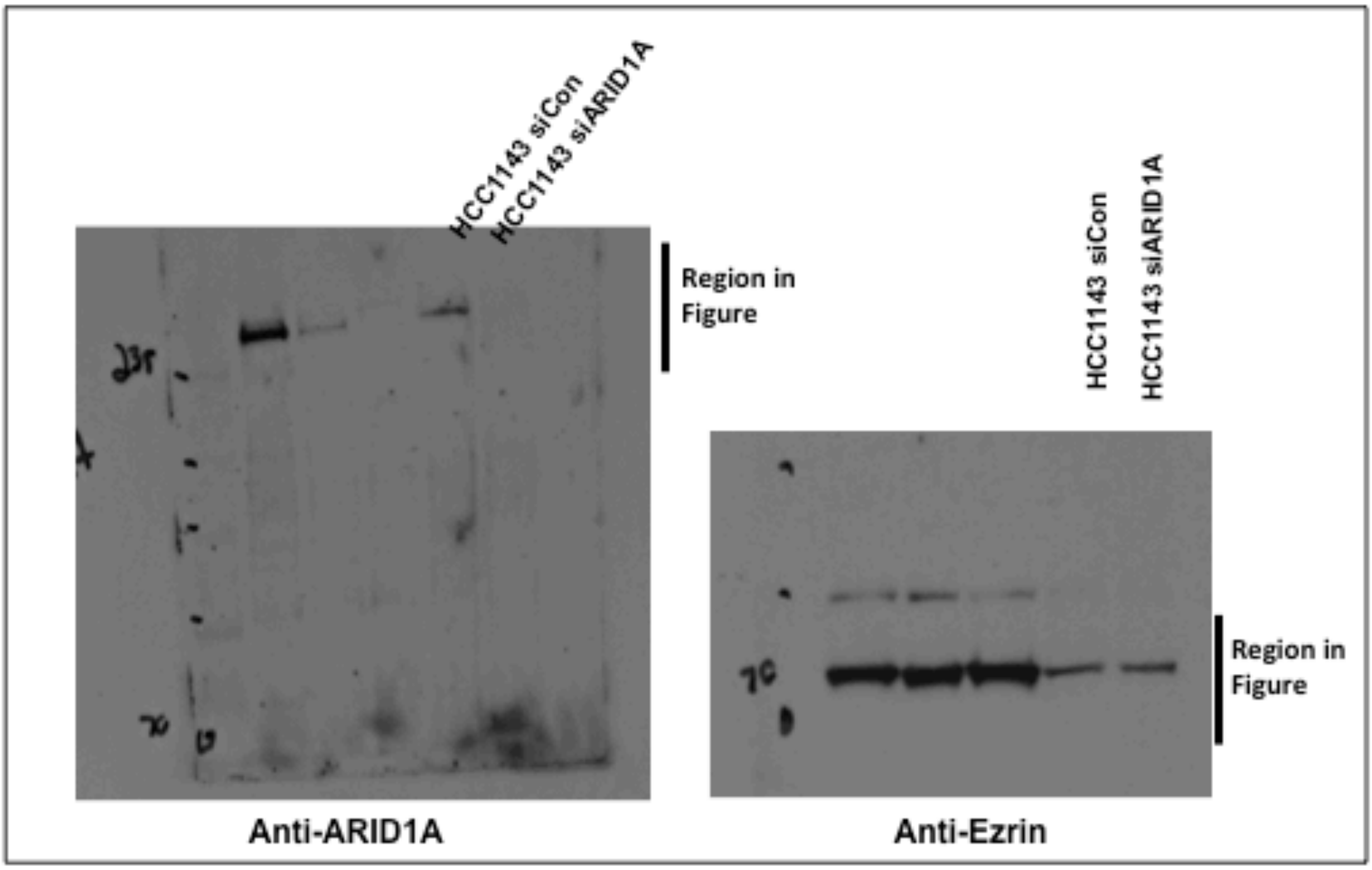

HCC1143 siCon
HCC1143 siARID1A
Region in Figure
70
Anti-ARID1A
HCC1143 siCon
HCC1143 siARID1A
70
Region in Figure
Anti-Ezrin

B

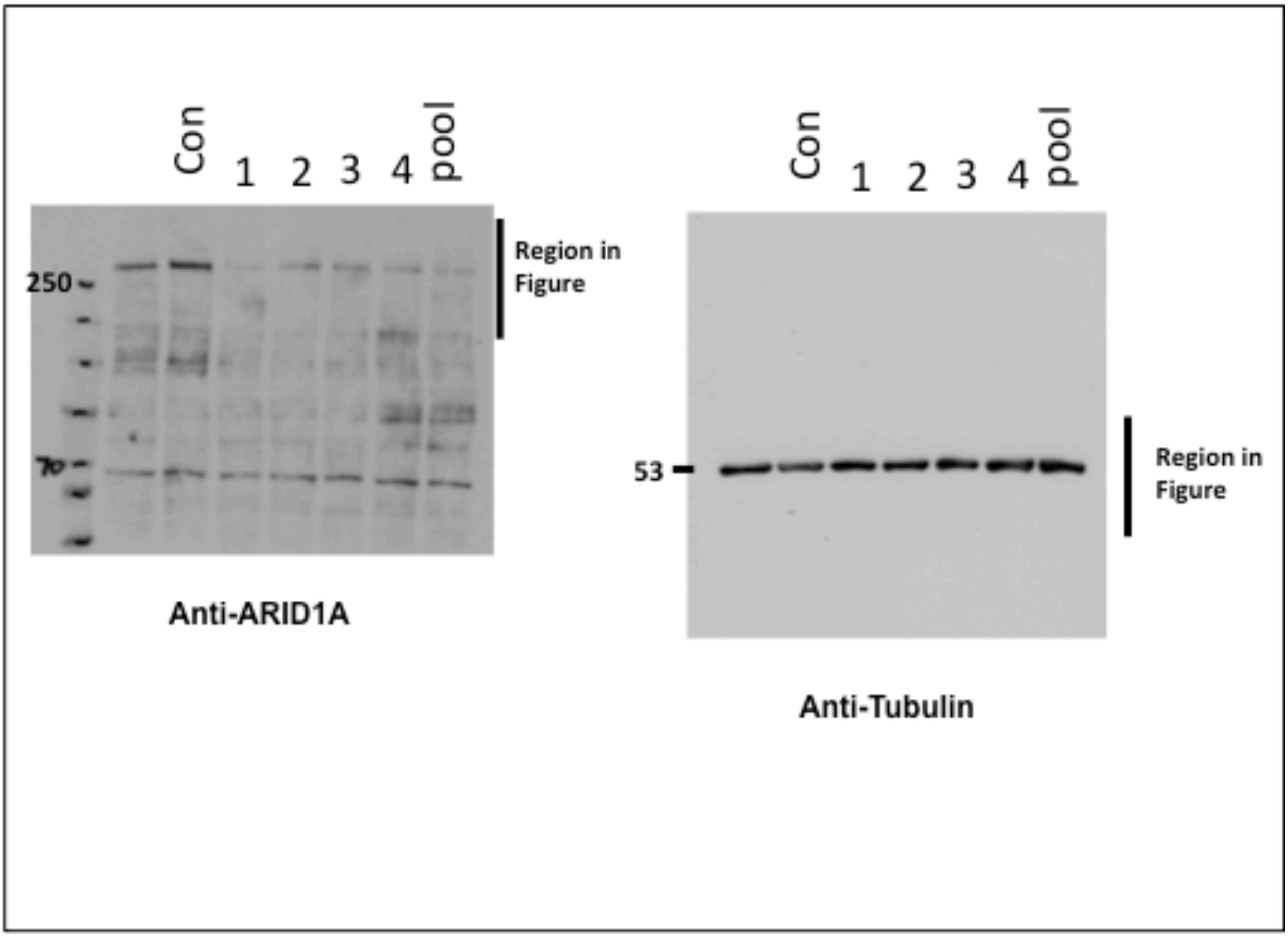

Con 1 2 3 4 pool
250
70
Region in Figure
Anti-ARID1A
Con 1 2 3 4 pool
53
Region in Figure
Anti-Tubulin

Supplementary Figure 6.

A. Uncropped scans of western blots presented in Figure 1F. B. Uncropped scans of western blots presented in Figure 1H.

A

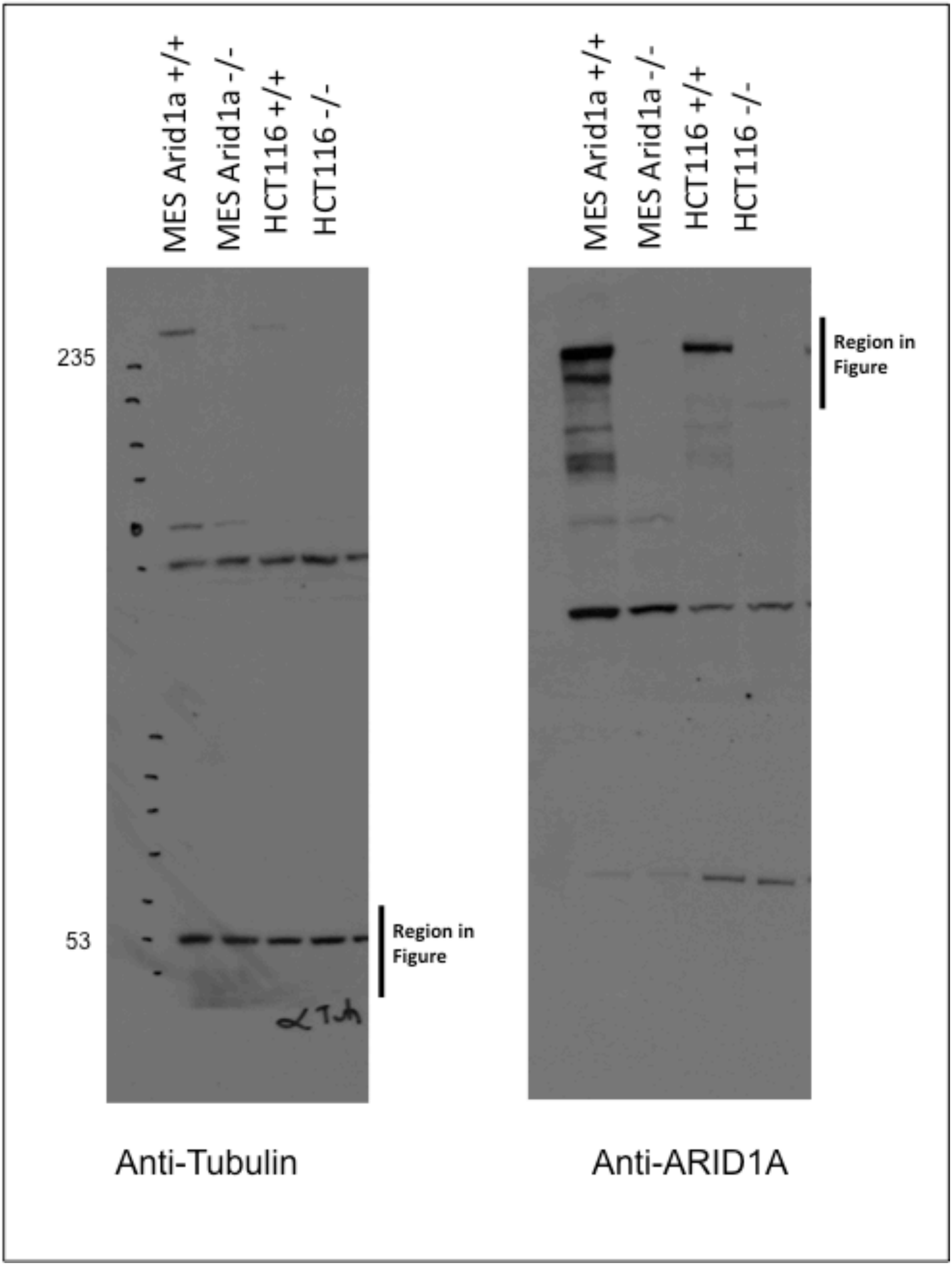
MES Arid1a +/+
MES Arid1a -/-
HCT116 +/+
HCT116 -/-
235
53
Region in Figure
αTub
Anti-Tubulin
MES Arid1a +/+
MES Arid1a -/-
HCT116 +/+
HCT116 -/-
Region in Figure
Anti-ARID1A

Supplementary Figure 7.
A. Uncropped scans of western blots presented in Figure 2B and 2L.

A

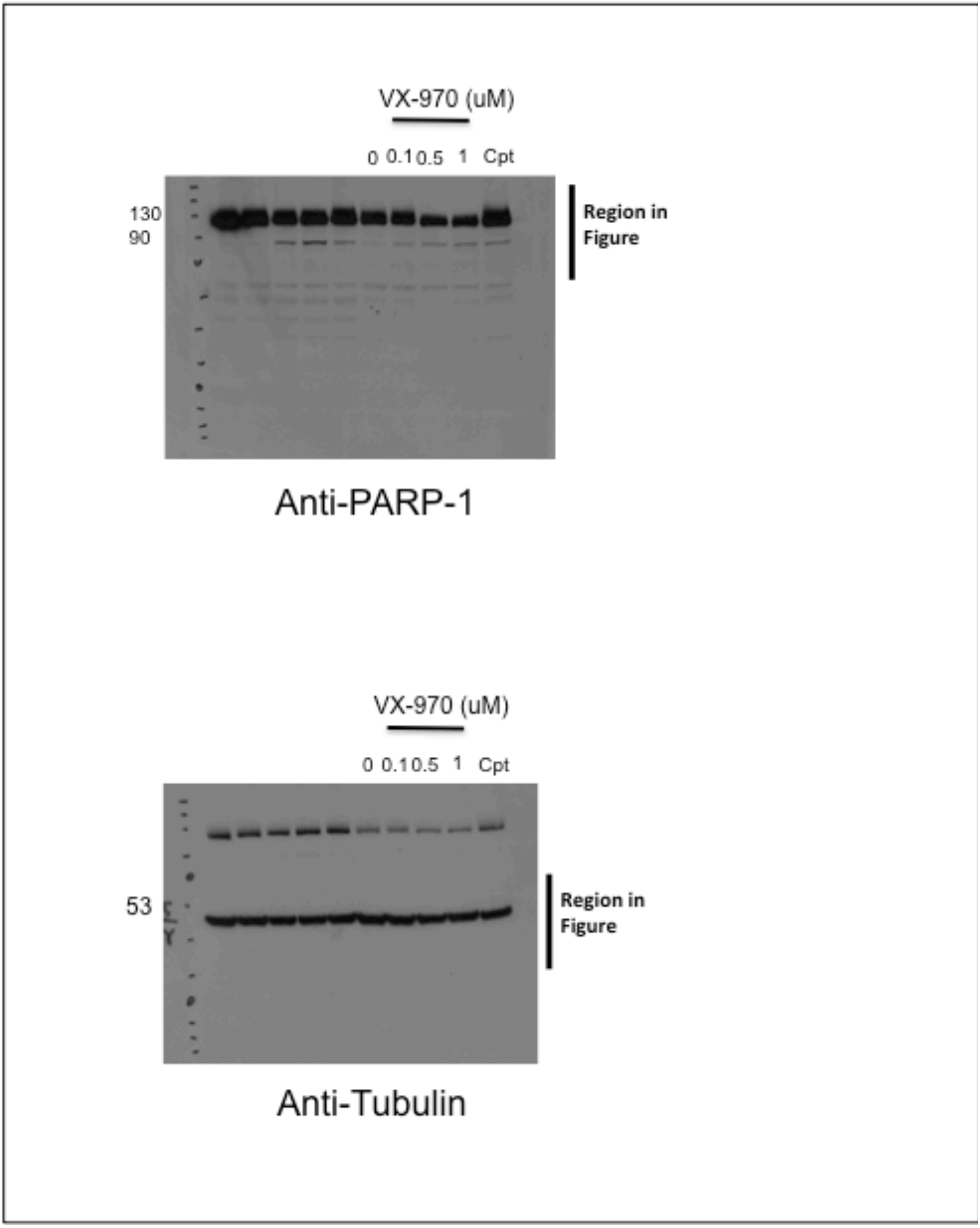

VX-970 (uM)
0 0.1 0.5 1 Cpt
130
90
Region in Figure
Anti-PARP-1
VX-970 (uM)
0 0.1 0.5 1 Cpt
53
Region in Figure
Anti-Tubulin

Supplementary Figure 8.

A. Uncropped scans of western blots presented in Figure 3H.

A

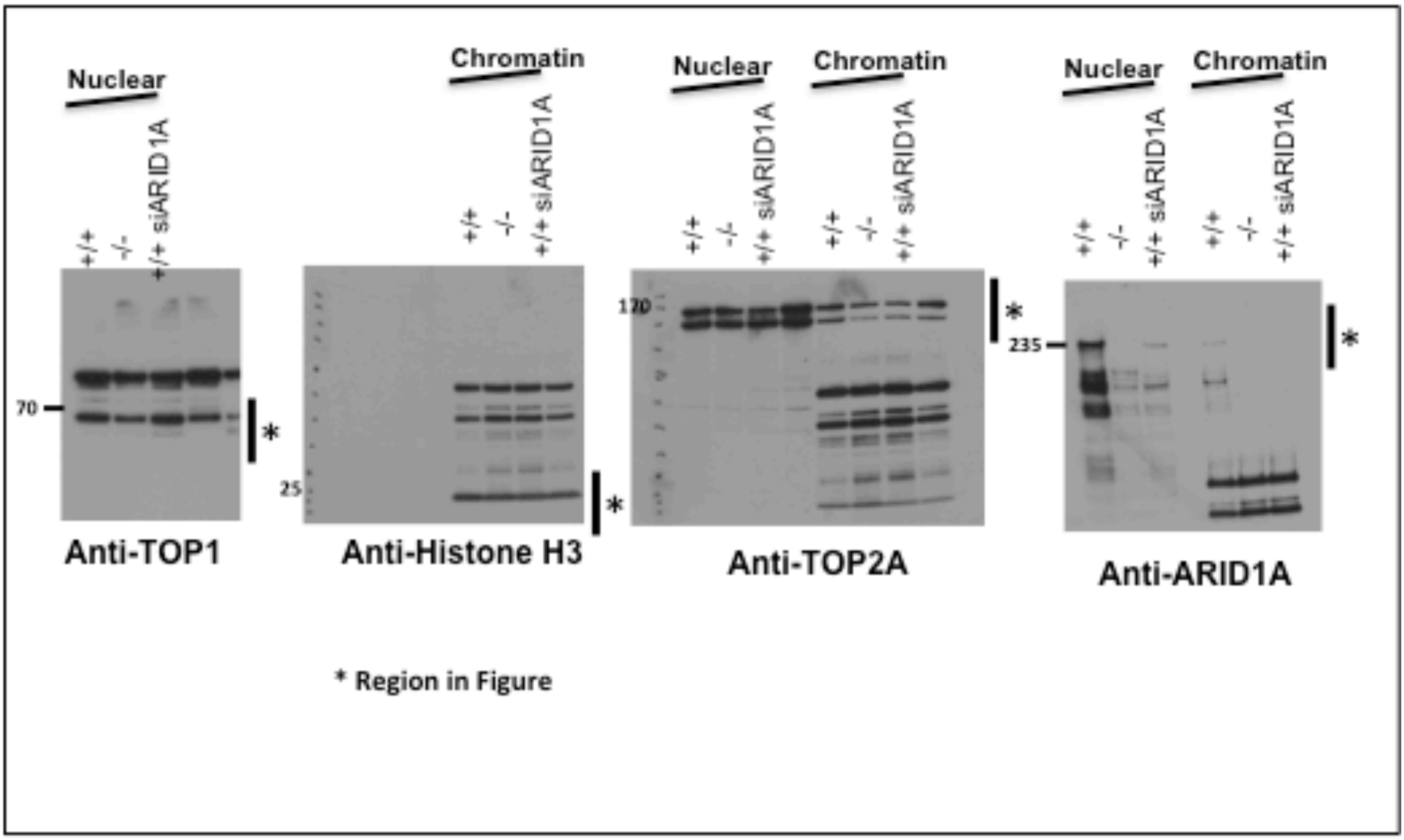
Nuclear
Chromatin
+/+
-/-
+/+ siARID1A
70
25
170
235
*
Anti-TOP1
Anti-Histone H3
Anti-TOP2A
Anti-ARID1A
* Region in Figure

B

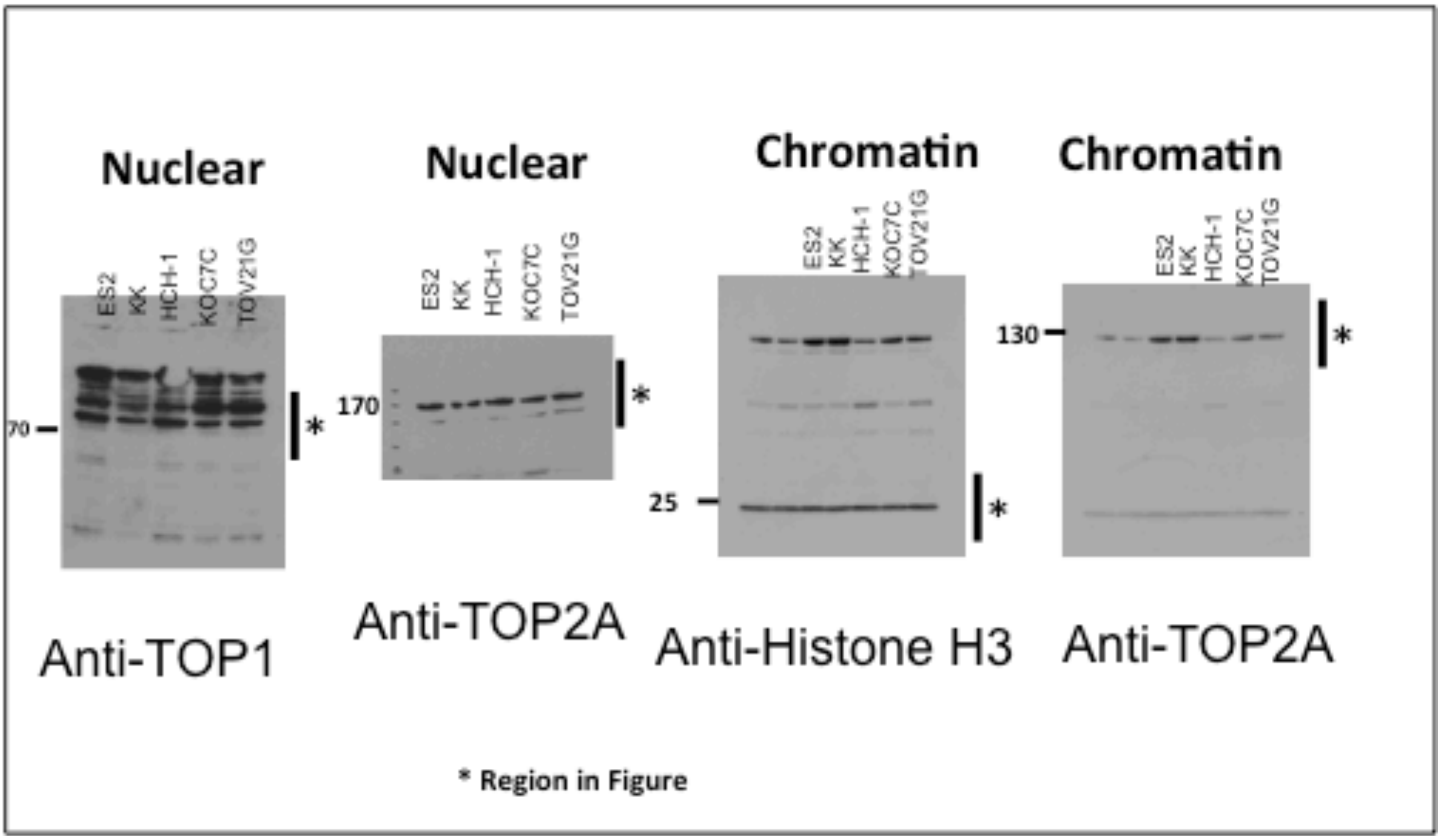
Nuclear
Nuclear
Chromatin
Chromatin
ES2
KK
HCH-1
KOC7C
TOV21G
70
170
25
130
*
Anti-TOP1
Anti-TOP2A
Anti-Histone H3
Anti-TOP2A
* Region in Figure

Supplementary Figure 9.
A. Uncropped scans of western blots presented in Figure 4D. B. Uncropped scans of western blots presented in Figure 4F.

A

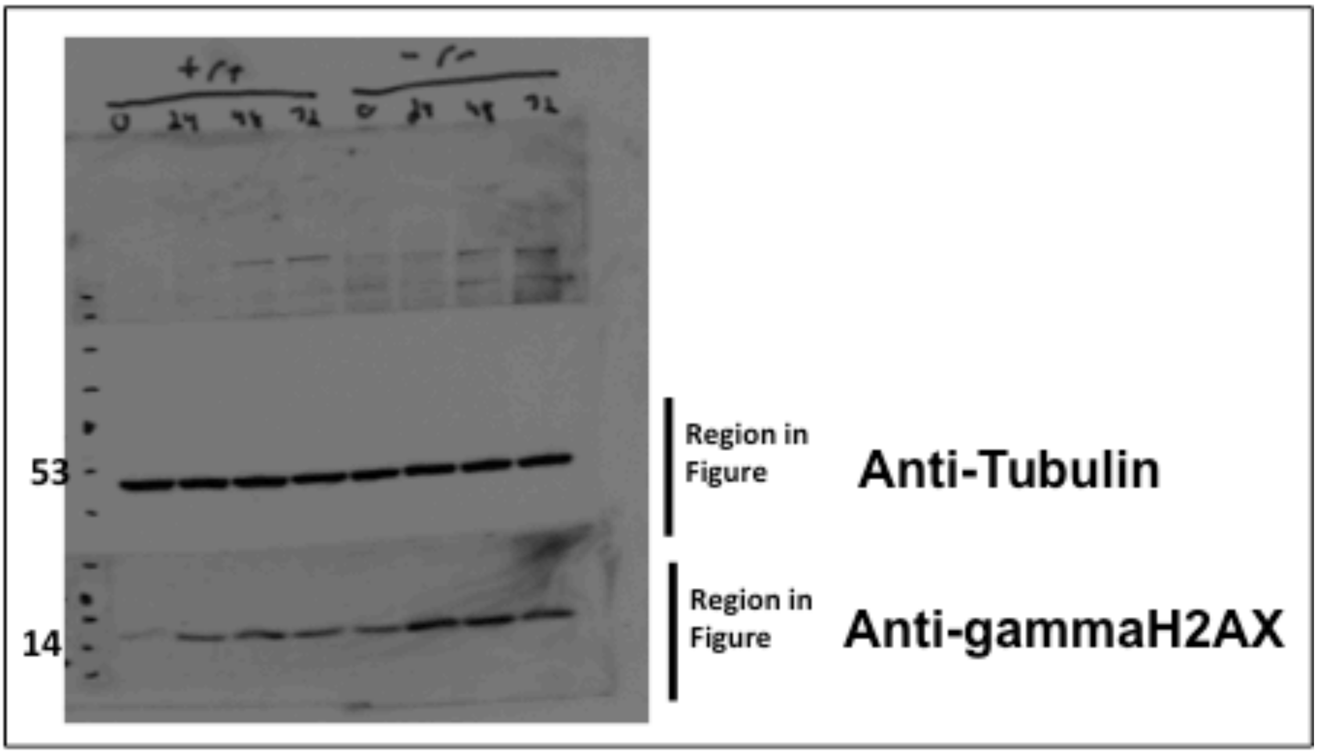
53
14
Region in Figure
Anti-Tubulin
Region in Figure
Anti-gammaH2AX

B

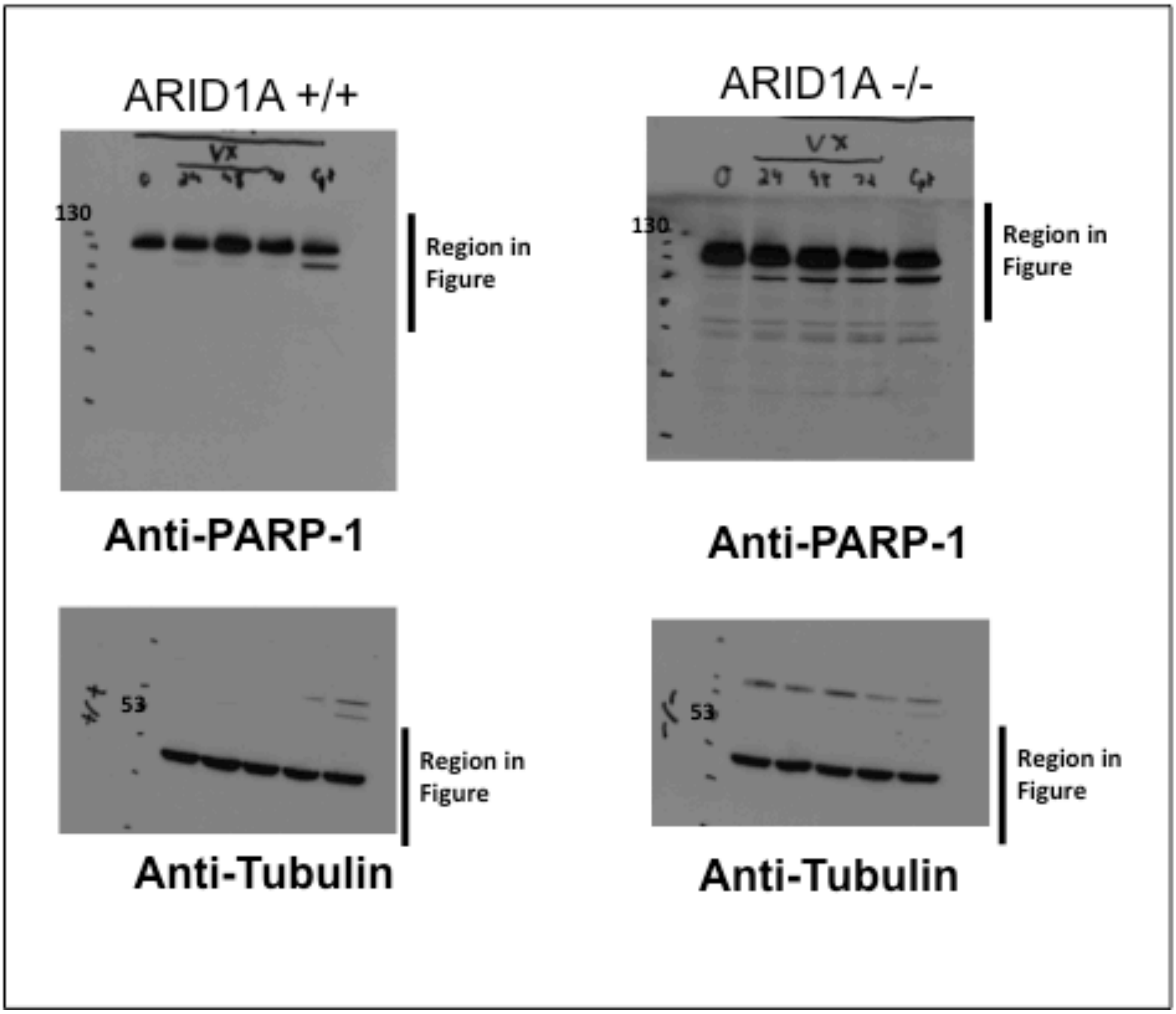
ARID1A +/+
ARID1A -/-
130
Region in Figure
130
Region in Figure
Anti-PARP-1
Anti-PARP-1
53
Region in Figure
53
Region in Figure
Anti-Tubulin
Anti-Tubulin

Supplementary Figure 10.
A. Uncropped scans of western blots presented in Figure 5H. B. Uncropped scans of western blots presented in Figure 5F.
